# ADHD as a Risk Factor for Infection With Covid-19

**DOI:** 10.1177/1087054720943271

**Published:** 2020-07-22

**Authors:** Eugene Merzon, Iris Manor, Ann Rotem, Tzipporah Schneider, Shlomo Vinker, Avivit Golan Cohen, Ari Lauden, Abraham Weizman, Ilan Green

**Affiliations:** 1 Leumit HMO, Tel-Aviv, Israel; 2 Tel Aviv University, Israel; 3 Geha Mental Health Center, Petah Tikva, Israel; 4 Clalit Health Services, Bnei Brak, Israel; 5 Felsenstein Medical Research Center, Petah Tikva, Israel

**Keywords:** ADHD, Covid-19, prevalence, stimulants

## Abstract

**Background:** ADHD limits the ability to comply with Covid-19 prevention recommendations. We hypothesized that ADHD constitutes a risk factor for Covid-19 infection and that pharmacotherapy may lower that risk. **Methods:** Study population included all subjects (*N* = 14,022) registered with Leumit Health Services between February 1st and April 30, 2020, who underwent at least one Covid-19 test. Data were collected from the electronic health records. Purchasing consecutively at least three ADHD-medication-prescriptions during past year was considered drug-treatment. **Results:** A total of 1,416 (10.1%) subjects (aged 2 months–103 years) were Covid-19-positive.They were significantly younger, and had higher rates of ADHD (adjOR 1.58 (95% CI 1.27–1.96, *p* < .001) than Covid-19-negative subjects. The risk for Covid-19-Positive was higher in untreated-ADHD subjects compared to non-ADHD subjects [crudeOR 1.61 (95% CI 1.36–1.89, *p* < .001)], while no higher risk was detected in treated ones [crudeOR 1.07 (95% CI 0.78–1.48, *p* = .65)]. **Conclusion:** Untreated ADHD seems to constitute a risk factor for Covid-19 infection while drug-treatment ameliorates this effect.

## Introduction

The coronavirus disease 2019 (COVID-19) pandemic has rapidly spread from Wuhan, China in late 2019 (Li et al., & Tang, 2020; Sohrabi et al., 2020) to nearly every country across the globe (World Health Organization, 2020a) and poses a global public health and economic threat (World Health Organization, 2020b; Wu et al., 2020). The severe acute respiratory syndrome coronavirus 2 (SARS-CoV-2) has high levels of transmissibility, estimated basic reproduction (R_o_) ranging from 2.6 to 4.7 and incubation duration ranging from 2 to 14 days (Lauer et al., 2020).

The main routes of transmission are respiratory droplets as well as direct contact with contaminated objects and surfaces (Van Doremalen et al., 2020). Based on the experience from China and information on viral transmission, early steps were taken in many countries including Israel to reduce the chance of being infected and reduce the spread of Covid-19 infection. These steps included governmental instructions designed to prevent the spread of the disease. Flights were cancelled and non-essential places of business were shut down by government orders. Various countries instituted a 14-day quarantine for those who were in close contact with Covid-19 verified patients or upon returning from epidemic countries (Israel Ministry of Health, 2020a; Lin et al., 2020).

Subsequently, people were asked to avoid close contact, maintaining a 2-m distance from each other (Centers for Disease Control and Prevention, 2020a; World Health Organization, 2020c). Wearing face masks in public was also requested as well as frequent hand washing. People were asked to stay home and self-isolate even with minor symptoms such as headache, fever, or a mild cough. These actions resulted in a significant reduction in the spread of the virus (Cheng et al., 2020; Lai et al., 2020).

ADHD is classified as a neurodevelopmental disorder. The DSM-5 diagnosis in the pediatric population requires the presence of ≥6 symptoms of inattention and/or ≥6 symptoms of hyperactivity/impulsivity (in adults, ≥5 symptoms of each), that have persisted for ≥6 months to a degree that is inconsistent with the developmental level and negatively impacts social and academic/occupational activities, and that in four conditions (American Psychiatric Association, 2013). The criteria include symptoms that tend to increase the risk for viral transmission. Relevant inattentive criteria include failure to give close attention to details or making careless mistakes, not seeming to listen when spoken to directly, not following through on instructions and being easily distracted, difficulty in keeping materials and belongings in order, and losing things necessary for tasks or activities. Additional pertinent hyperactive-impulsive criteria include fidgeting with or tapping hands or feet, or squirming in seat, leaving seat in situations when remaining seated is expected, taking risks, and restlessness. Thus, it is expected that the complex of inattention, hyperactivity, and impulsivity would tend to increase the probability of exposure to Sars-Cov-2.

The basic requirements of physical distancing, avoiding touch, washing hands frequently and intensively, and wearing a face mask in public, as well as staying at home, self-isolation, and maintaining quarantine are all more difficult to follow for people who live with ADHD. This is especially true for those with the combined and the hyperactive presentations, compared to people without ADHD. Such ADHD-related difficulties limit the ability to comply with the basic recommendations for the prevention of Covid-19, particularly in younger age groups.

### Hypotheses

The hypotheses of this study were that the rate of ADHD among Covid-19 positive subjects is significantly higher compared to those without ADHD and that treatment with anti-ADHD medications may moderate the rate of Covid-19 infection.

## Methods

Leumit Health Services (LHS) has a comprehensive computerized database, continuously updated with regard to a subjects’ demographics, medical visits, laboratory tests, hospitalizations, and medication prescriptions. Prescription records are available starting from 1998 and include those refilled and purchased per patient. All study participants have similar health insurance and similar access to health services. During each physician visit, a diagnosis is established according to the International Classification of Diseases-9/10 (ICD-9 or ICD-10), depending on the date of diagnosis. The validity of the diagnoses from electronic medical records was previously estimated in several studies and found to be high for chronic diagnoses and laboratory data (Pringle et al., 1995; Shalev et al., 2011).

The study period was from February 1st to April 30th 2020. The study population consisted of all subjects who were registered with LHS during the study period and underwent at least one test for Covid-19. The Covid-19 test used in Israel is the 2019-nCoV RT-PCR diagnostic panel the AllplexTM 2019-nCoV Assay (Seegene Inc., Seoul, Republic of Korea) (World Health Organization, 2020d). The referral for the Covid-19 test was either made by their physician or emergency medical services according to the Israeli Ministry of Health’s guidelines for symptoms and close contact with Covid-19 positive subjects.

Data were collected from the LHS computerized database and include demographics for the entire cohort. Socioeconomic status (SES) was defined according to the child’s home address, using the Israeli Central Bureau of Statistics’ classification which includes 20 subgroups. Classifications one to nine were considered low-medium SES, and 10 to 20 were considered medium-high SES. The data also included smoking and obesity status, and other relevant diagnoses such as: asthma, COPD, diabetes, hypertension as well as other chronic medical diagnoses (including somatic and psychiatric disorders).

ADHD was diagnosed according to the Israeli Ministry of Health criteria, which follows the international requirements for evaluation. The diagnosing physician must be a senior physician, who has a specialty in the ADHD field (child or adult psychiatrists, child or adult neurologists, or pediatricians and family physicians with certified ADHD training). The diagnosis is established according to the American Psychiatric Association’s Diagnostic and Statistical Manual (DSM-IV or 5, depending on the year of the diagnosis) criteria (American Psychiatric Association, 2013).

We hypothesized that the risk of being infected by Covid-19 may be affected not only by having ADHD diagnosis, but also by the treatment status.

Subjects, who purchased at least three consecutive prescriptions of ADHD medication during the past 12 months were defined as medically-treated ADHD subjects. The most prevalent medications were stimulants (92.9% stimulants, 7.1% non-stimulants), hence we referred to the medications as stimulants.

All the psychiatric diagnoses were based on the International Classification of Disease, ninth revision (ICD-9) or 10th revision (ICD-10) codes, as is required by all Israeli Health Maintenance Organizations (HMOs). The psychiatric diagnoses in the electronic health records, in addition to ADHD, included autism spectrum disorder (ASD), depressive disorders, anxiety disorders, schizophrenia, and dementia.

The study protocol was approved by the Shamir Medical Center Review Board and the Research Committee of LHS.

### Statistical Analysis

Statistical analysis was conducted using STATA 12 software (StataCorp LP, College Station, TX). Assumptions were two sided with an α of .05. Initial analysis compared demographic characteristics between the study groups (positive vs. negative Covid-19 tests), using Student’s *t*-test and Fisher’s exact χ^2^ test for continuous and categorical variables, respectively, based on normal distribution and variable characteristics. Categorical data are shown in counts and percentages. Data on continuous variables with normal distribution are presented as mean and 95% confidence interval (95% CI).

Preliminary evaluation of risk estimates was conducted by stratified analyses. Subsequently, multivariate logistic regression was used to estimate the odds ratios (OR) and 95% CI for the independent association between ADHD and positive PCR test for Covid-19 while controlling for potential confounders.

## Results

### Whole Sample: Univariate Analysis of Covid-19-Positive Versus Covid-19-Negative

This study included 14,022 subjects, aged from 2 months old to 103 years old, who were tested for Covid-19. Among them 1,416 (10.1%) had at least one positive result, and 12,606 (89.9%) had only negative results. In a primary univariate analysis, Covid-19-positive (Covid-19-P) subjects were younger, more likely to be males, and belonged to a lower SES than Covid-19-negative (Covid-19-N) subjects (Table 1).

**Table 1.** Demographic Characteristics of the Study Sample Stratified by COVID-19 Tests Results.

Demographics	Total	Covid-19 positive *n* = 1,416 (10.1%)	Covid-19 negative *n* = 12,606 (89.9%)	*p* value
Mean age (years, 95% CI)	38.94 (38.57–39.29)	35.58 (34.49–36.67)	39.31 (38.92–39.70)	<.001
Age categories *N* (%)				
<20 years	2,424 (17.29%)	366 (25.85%)	2,058 (16.33%)	<.001
21–40 years	5,559 (39.64%)	488 (34.46%)	5,071 (40.23%)	.065
41–60 years	3,379 (24.10%)	349 (24.65%)	3,030 (24.05%)	.082
>60 years	2,660 (18.97%)	213 (15.04%)	2,447 (19.39%)	.054
SES *N* (%)				
Low	9,295 (72.24%)	1,115 (78.74%)	8,180 (64.89%)	<.001
High	3,571 (27.76%)	191 (13.49%)	3,380 (26.81%)	<.001
Gender *N* (%)				
Male	7,340 (52.35%)	789 (55.72%)	6,551 (51.97%)	<.001
Female	6,682 (47.65%)	627 (44.28%)	6,055 (48.03%)	<.001

*Note*. CI = confidence interval; SES = socioeconomic status.

The medical conditions of the sample exhibited several significant differences between the Covid-19-P and the Covid-19-N subjects (Table 2). The most prominent ones were the presence of ADHD (*p* < .001), dementia (*p* < .001), hypertension (*p* < .001), and chronic lung disease (*p* < .001) (Table 2).

**Table 2.** Psychiatric and Somatic Comorbidities of the Study Sample Stratified by COVID-19 Tests Results.

Variable	Total	Covid-19 positive *n* = 1,416 (10.1%)	Covid-19 negative *n* = 12,606 (89.9%)	*p* value
ADHD *N* (%)	1,699 (12.12%)	230 (16.24%)	1,469 (11.65%)	<.001
ASD *N* (%)	53 (0.38%)	5 (0.35%)	48 (0.38%)	.872
Depression and anxiety *N* (%)	1,155 (8.24%)	94 (6.64%)	1,061 (8.42%)	.042
Schizophrenia *N* (%)	264 (1.88%)	26 (1.84%)	238 (1.89%)	.892
Dementia *N* (%)	542 (3.87%)	32 (2.26%)	510 (4.05%)	<.001
Obesity *N* (%)	2,963 (25.18%)	307 (26.31%)	2,656 (25.06%)	.350
Diabetes mellitus *N* (%)	2,094 (14.94%)	183 (12.96%)	1,911 (15.16%)	.003
Hypertension *N* (%)	2,628 (18.74%)	199 (14.05%)	2,429 (19.27%)	<.001
Chronic lung disease *N* (%)	1,512 (10.78%)	101 (7.13%)	1,411 (11.19%)	<.001
Smoking^a^ *N* (%)	2,296 (16.37%)	254 (17.94%)	2,042 (16.20%)	.099

*Note*. ASD = autism spectrum disorder; Chronic lung disease = asthma and COPD.

a 28.55% missing data.

In the univariate analysis, the likelihood of Covid-19 infection was positively associated with having a diagnosis of ADHD [crude OR of 1.47 (95% CI 1.26–1.71, *p* < .001)], being younger than 20 years old [crude OR of 1.78 (95% CI 1.57–2.03, *p* < .001)], being male [crude OR of 1.16 (95% CI 1.04–1.29, *p* < .05)] and being part of the low-medium SES group [crude OR of 2.41 (95% CI 2.06–2.83, *p* < .001)]. The likelihood of Covid-19 infection was negatively associated with having a diagnosis of depression/anxiety [crude OR of 0.77 (95% CI 0.62–0.96, *p* < .001)], dementia [crude OR of 0.55 (95% CI 0.38–0.79, *p* < .001)], diabetes mellitus [crude OR of 0.83 (95% CI 0.71–0.98, *p* < .001)], hypertension [crude OR of 0.83 (95% CI 0.71–0.98, *p* < .001)] and chronic lung disease [crude OR of 0.61 (95% CI 0.49; 0.75, *p* < .001)] (Table 3). After controlling the demographic and the medical variables in a multivariate analysis, the adjusted odds ratio of ADHD increased to 1.58 (95% CI 1.27–1.96, *p* < .001), emphasizing the significance of the association between ADHD and Covid-19. The positive association of Covid-19 infection with male gender, age below 20 years and low-medium SES group remained significant, adjusted OR of 1.18 [(95% CI 1.01–1.37, *p* < .05)] 2.08 [(95% CI 1.61–2.68, *p* < .001)], and 1.96 [(95% CI 1.63–2.36, *p* < .001)], respectively. The negative association of Covid-19 infection remained significant only with having a chronic lung disease [adjusted OR of 0.58 (95% CI 0.45–0.76, *p* < .001] (Table 3).

**Table 3.** Odds Ratios of the Probability of the Various Clinical Diagnoses of the Whole Sample to be Associated With Covid-19 Infection.

Variable	Crude OR (95% CI)	*p* value	Adjusted OR (95% CI)	*p* value
ADHD	1.47 (1.26–1.71)	<.001	1.58 (1.27–1.96)	<.001
Age <20 years	1.79 (1.57–2.03)	<.001	2.08 (1.61–2.68)	<.001
Male gender	1.16 (1.04–1.29)	<.001	1.18 (1.01–1.37)	<.001
Low-medium SES	2.41 (2.06–2.83)	<.001	1.96 (1.63–2.36)	<.001
Obesity	1.07 (0.93–1.23)	.350	1.12 (0.95–1.32)	.177
ASD	0.93 (0.37–2.33)	.872	1.39 (0.40–4.85)	.603
Depression/anxiety	0.77 (0.62–0.96)	.008	1.09 (0.84–1.40)	.528
Schizophrenia	0.97 (0.65–1.46)	.892	1.21 (0.75–1.96)	.439
Dementia	0.55 (0.38–0.79)	<.001	0.65 (0.39–1.07)	.087
Diabetes mellitus	0.83 (0.71–0.98)	.042	1.00 (0.80–1.25)	.986
Hypertension	0.69 (0.59–0.81)	<.001	0.83 (0.66–1.04)	.103
Chronic lung disease	0.61 (0.49–0.75)	<.001	0.58 (0.45–0.76)	<.001
Smoking	1.13 (0.98–1.31)	.099	0.95 (0.74–1.16)	.075

*Note*. OR = odds ratio; CI = confidence interval; SES = socioeconomic status; ASD = autism spectrum disorder; chronic lung disease = asthma and COPD.

Among the 1,699 subjects with a diagnosed ADHD, 418 (24.6%) have purchased during the last 12 months at least three prescriptions of ADHD medications and were defined as treated. About 92% of the medications were stimulants.

In order to clarify the effect of ADHD pharmacotherapy, the sample was stratified to three strata: non-ADHD, treated ADHD, and untreated ADHD. The subjects with treated ADHD had no additional risk of Covid-19 [crude OR of 1.07 (95% CI 0.78; 1.48, *p* = .65)], compared to non-ADHD subjects (a reference group), while the risk of subjects with untreated ADHD was significantly increased [crude OR of 1.61 (95% CI 1.36–1.89, *p* < .001)] (Table 4).

**Table 4.** ORs of the Whole Sample to be Infected by Covid-19 Stratified by Having ADHD and Being Treated.

Variable	Covid-19 positive *n* = 1,416 (10.1%)	Covid-19 negative *n* = 12,606 (89.9%)	Crude OR (95% CI)	Adjusted OR (95% CI)	*p* value
Non-ADHD	1,186 (83.76%)	11,137 (88.35%)	1 (reference group)	1 (reference group)	
ADHD-treated^a^	43 (3.04%)	375 (2.97%)	1.07 (0.78–1.48)	0.85 (0.54–1.34)	.707
ADHD-untreated	187 (13.21%)	1,094 (8.68%)	1.61 (1.36–1.89)	1.68 (1.37–2.10)	<.001

*Note.* CI = confidence interval.

a Treated ADHD—ADHD treated with pharmacotherapy.

**Figure 1.**
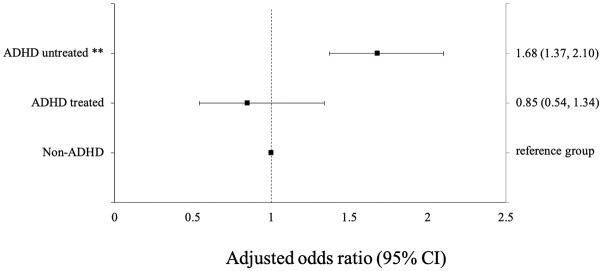
Adjusted odds ratios (error bars indicate 95% confidence intervals) for being Covid-19 positive in ADHD treated and untreated patients. Note. Treated ADHD—ADHD treated with pharmacotherapy. CI = confidence interval. **p < .001.

After an adjustment for possible confounders, the association between ADHD treatment and the risk of infection with Covid-19 became more prominent for untreated ADHD versus treated ADHD [adjusted OR 1.68 (95% CI 1.37–2.10, *p* < .001 vs. adjusted OR 0.85 (95% CI 0.54–1.34, *p* = .707), respectively] (Table 4, Figure 1).

### Sensitivity Analysis in Subjects Aged 5 to 20 Years

A selected group of subjects aged 5 to 20 years, ages in which ADHD diagnosis is more prevalent, was analyzed separately, as a sensitivity analysis. In this restricted population the ADHD prevalence became larger, both among Covid-19-P and Covid-19-N patients (28.14% vs. 18.85%, *p* < .001), with increasing odds of the ADHD subjects to be infected with Covid-19 [adjusted OR 1.92 (95% CI 1.46–2.53, *p* < .001)].

### The Impact of Medications Within the ADHD Group

In order to strengthen the effect of pharmacotherapy on Covid-19, an analysis of the ADHD population, treated versus untreated was performed separately. The group of treated ADHD subjects was younger, 22.6 years (95% CI 21.4–23.7) versus 26.2 years (95% CI 25.6–26.7) but there was no other significant difference in any demographic variable.

Treated ADHD subjects had a significantly lower likelihood to be infected by Covid-19, as compared to the untreated ones [adjusted OR of 0.63 (95% CI 0.42–0.94 *p* < .001)] (Table 4, Figure 1).

## Discussion

Vulnerability to Covid-19 infection is still not well understood and the information about it is scarce, thus the best way to tackle it is through a focus on high-risk groups. This study examined the possible risk factors, from demographic ones, as age and gender, to the medical ones. The studied population included all the suspected subjects of an Israeli HMO in the first 2 months of the epidemic, who were tested for Covid-19, and the infection rate was found to be 10.1%. It is interesting to note that the Covid-19 positive patients tended to be younger, to be males and from a lower SES group. The tendency toward a lower SES group could be explained by the higher density of the population in this strata. These findings are consistent with other urban reports such as in New York City where there was a higher likelihood for a positive test amongst those who live in lower SES neighborhoods and those who live in larger households (Borjas, 2020).

The independent association between young age and increased likelihood of Covid-19 infection early in Israel’s Covid-19 outbreak has been poorly reported elsewhere due to lack of early widespread testing. However, a similar distribution amongst young adults has been reported in South Korea, a country who also applied early vigorous testing (Shim et al., 2020). Similarly, the initial low case fatality rate in England may have been a reflection of the relatively young age structure of early infections (Dowd et al., 2020). This association may be related to risky behaviors such as the attendance of mass gatherings as well as the tendency of this age group to cling together, remaining non-compliant with social distancing measures. For instance, in Korea’s recent Covid-19 “second wave,” hundreds of infections have been linked to nightclubs and other entertainment venues, which saw large crowds in early May after officials relaxed social distancing guidelines (Gang-lip, 2020). Moreover, an adjustment for age younger than 20 years old in a multivariate logistic regression analysis increased the odds ratio of association between the previous diagnosis of ADHD and the risk of being infected with Covid 19.

Medical conditions, like diabetes, hypertension, and chronic lung diseases that were considered to be associated with increased rate of fatality (Docherty et al., 2020) were not linked to an elevated rate of infection in this study. This is most probably due to the strict “stay at home” restrictions that were particularly stressed in this vulnerable population (Centers for Disease Control and Prevention, 2020b; Israel Ministry of Health, 2020b).

On the other hand, mental health problems were not identified as possible risk factors for Covid-19 infection. It was found that in contrast to all the other mental health disorders that were assessed, only the rate of ADHD was significantly higher among the Covid-19-P subjects, irrespective of age, gender, SES and the presence of other chronic mental and physical disorders. This finding is especially robust, since most of the diagnosed ADHD patients are children (Ginsberg et al., 2014a, 2014b), and that the pediatric population accounted for less than 2% of Covid-19 identified cases during the same time period (Centers for Disease Control and Prevention, 2020c; Lu et al., 2020). Thus, it seems that ADHD by itself is a plausible risk factor for this infection.

One possible explanation of the significant vulnerability of ADHD subjects to be infected by Covid-19 may be ascribed to the inattentive, hyperactive, and impulsive characteristics of ADHD. These characteristics of ADHD interfere with the ability to comply with WHO demands for the prevention of Covid-19 infection. Interestingly, other tested psychiatric disorders did not have the same effect on the rate of Covid-19 infection. Noteworthy, schizophrenia that shares some of the clinical characteristics of ADHD such as distractibility, inattention in addition to impaired judgment, was not associated with significant elevated risk for infection with Covid-19 (Table 3). Another characteristic of people with ADHD is their difficulty in taking orders and to be disciplined. Thus, it may be that their compliance to the restrictions that were imposed on the population was lower, thus increasing their vulnerability to be infected.

In addition, the rate of Covid-19-P tests was significantly higher in untreated ADHD subjects, while in ADHD subjects treated with pharmacotherapy the rate was even lower than that of non-ADHD subjects. This finding indicates the possible protective role of pharmacotherapy in the attenuation of Covid-19 transmission.

The notion of dysregulated behavior as a cause for increased risk for infection with Covid-19 is supported by the reduced rate of infection in ADHD treated patients, as compared to the higher rate of infection in the untreated ones. Therefore, it is suggested that physicians will educate parents, as well as young and adult ADHD patients on the potential for increased risk, as well as risk amelioration through medication.

### Strengths and Weaknesses

The main strengths of the study were its large size, and its real-world, population-based nature. An additional strength is the inclusion of a multitude of variables that may affect the likelihood for infection with Covid-19, independently of ADHD, which have not been previously been examined before in such detail.

However, there were major weaknesses of the study, especially its retrospective database design. Furthermore, relevant clinical variables such as the severity of ADHD symptoms, or its presentation (predominantly inattentive, predominantly hyperactive or combined) were not available in the electronic recorded data.

In addition, data regarding the presenting symptoms and severity of Covid-19 infection, as well as adverse clinical outcomes (hospitalization and mechanical ventilation) were not assessed.

In conclusion, this study found that ADHD may be a risk factor for Covid-19 infection, independently of other risk factors. The pharmacotherapy of ADHD (mostly stimulants) appeared to moderate the risk of infection. This is an important finding, since it could encourage healthcare systems to identify this population at risk, to increase their awareness to the necessary preventive actions, and to guide their parents, teachers, or personal caretakers to monitor carefully their behavior and the Covid-19 status. Adherence to anti-ADHD treatment should be encouraged in an attempt to reduce the spread of Covid-19 infection (Cortese et al., 2020). Large-scale replicative studies in non-Israeli populations, including assessments of the severity of ADHD symptoms, the pharmacological status and the presence of comorbid disorders that are common in ADHD, such as oppositional defiant disorder, conduct disorders, tic disorders, learning disabilities, and substance use disorders are warranted.

All the data related to this study can be accessed following approval of Leumit health services.

## References

[bibr1-1087054720943271] American Psychiatric Association. (2013). Diagnostic and statistical manual of mental disorders (DSM-5^®^). American Psychiatric Pub.

[bibr2-1087054720943271] Borjas G. J. (2020, April). Demographic determinants of testing incidence and COVID-19 infections in New York City neighborhoods (No. 26952). National Bureau of Economic Research.

[bibr3-1087054720943271] Centers for Disease Control and Prevention. (2020a, May 6). Social distancing. https://www.cdc.gov/coronavirus/2019-ncov/prevent-getting-sick/social-distancing.html (accessed 8 May 2020).

[bibr4-1087054720943271] Centers for Disease Control and Prevention. (2020b, May 14). People who are at higher risk for severe illness. https://www.cdc.gov/coronavirus/2019-ncov/need-extra-precautions/people-at-higher-risk.html (accessed 5 June 2020).

[bibr5-1087054720943271] Centers for Disease Control and Prevention. (2020c, April 10). Coronavirus disease 2019 in children—United States, February 12–April 2, 2020. Morbidity and Mortality Weekly Report, 69(14), 422–426. 10.15585/mmwr.mm6914e4 (accessed 5 June 2020). 32271728 PMC7147903

[bibr6-1087054720943271] Cheng V. C. Wong S. C. Chuang V. W. So S. Y. Chen J. H. Sridhar S. To K. K.-W. Chan J. F.-W. Hung I. F.-N. Ho P.-L. Yuen K. Y. (2020). The role of community-wide wearing of face mask for control of coronavirus disease 2019 (COVID-19) epidemic due to SARS-CoV-2. Journal of Infection, 81(1), 107–114. 10.1016/j.jinf.2020.04.024 PMC7177146 32335167

[bibr7-1087054720943271] Cortese S. Coghill D. Santosh P. Hollis C. Simonoff E. (2020). Starting ADHD medications during the COVID-19 pandemic: Recommendations from the European ADHD Guidelines Group. The Lancet Child & Adolescent Health, 4(6), 15. 10.1016/S2352-4642(20)30144-9 PMC7217636 32405517

[bibr8-1087054720943271] Docherty A. B. Harrison E. M. Green C. Hardwick H. E. Pius R. Norman L. Holden K. A. Read J. M. Dondelinger F. Carson G. Merson L. Lee J. Plotkin D. Sigfrid L. Halpin S. Jackson C. Gamble C. Horby P. W. Nguyen-Van-Tam J. S.. . . Semple M. G. (2020). Features of 20 133 UK patients in hospital with covid-19 using the ISARIC WHO Clinical Characterisation Protocol: Prospective observational cohort study. British Medical Journal, 369, m1985. 32444460 10.1136/bmj.m1985 PMC7243036

[bibr9-1087054720943271] Dowd J. B. Andriano L. Brazel D. M. Rotondi V. Block P. Ding X. Liu Y. Mills M. C. (2020). Demographic science aids in understanding the spread and fatality rates of COVID-19. Proceedings of the National Academy of Sciences of the United States of America, 117(18), 9696–9698. 32300018 10.1073/pnas.2004911117 PMC7211934

[bibr10-1087054720943271] Gang-lip K. (2020, May 28). Restrictions return in South Korea after new spike in COVID-19 cases. Euronews. https://www.euronews.com/2020/05/28/coronavirus-restrictions-return-in-south-korea-after-new-spike-in-covid-19-cases

[bibr11-1087054720943271] Ginsberg Y. Beusterien K. M. Amos K. Jousselin C. Asherson P. (2014a). The unmet needs of all adults with ADHD are not the same: A focus on Europe. Expert Review of Neurotherapeutics, 14(7), 799–812. 24894408 10.1586/14737175.2014.926220

[bibr12-1087054720943271] Ginsberg Y. Quintero J. Anand E. Casillas M. Upadhyaya H. P. (2014b). Underdiagnosis of attention-deficit/hyperactivity disorder in adult patients: A review of the literature. The Primary Care Companion for CNS Disorders, 16(3), PCC.13r01600. 10.4088/PCC.13r01600 PMC4195639 25317367

[bibr13-1087054720943271] Israel Ministry of Health. (2020a, January 24). Coronavirus: Avoid non-essential travel to Wuhan, China (press release). https://www.gov.il/he/departments/news/1_24012020 (accessed 8 May 2020).

[bibr14-1087054720943271] Israel Ministry of Health. (2020b, March 10). New Coronavirus guidance. https://www.health.gov.il/English/News_and_Events/Spokespersons_Messages/Pages/10032020_22.aspx (accessed 8 May 2020).

[bibr15-1087054720943271] Lai S. Ruktanonchai N. W. Zhou L. Prosper O. Luo W. Floyd J. R. Wesolowski A. Santillana M. Zhang C. Du X. Yu H. Tatem A. J. (2020). Effect of non-pharmaceutical interventions to contain COVID-19 in China. Nature. Advance online publication. 10.1038/s41586-020-2293-x PMC7116778 32365354

[bibr16-1087054720943271] Lauer S. A. Grantz K. H. Bi Q. Jones F. K. Zheng Q. Meredith H. R. Azman A. S. Reich N. G. Lessler J. (2020). The incubation period of coronavirus disease 2019 (COVID-19) from publicly reported confirmed cases: Estimation and application. Annals of Internal Medicine, 172(9), 577–582. 10.7326/M20-0504 32150748 PMC7081172

[bibr17-1087054720943271] Li H. Liu S. M. Yu X. H. Tang S. L. Tang C. K. (2020). Coronavirus disease 2019 (COVID-19): Current status and future perspective. International Journal of Antimicrobial Agents, 55(5), 105951. 32234466 10.1016/j.ijantimicag.2020.105951 PMC7139247

[bibr18-1087054720943271] Lin C. Braund W. E. Auerbach J. Chou J. H. Teng J. H. Tu P. Mullen J. (2020). Policy decisions and use of information technology to fight 2019 novel coronavirus disease, Taiwan. Emerging Infectious Diseases, 26(7), 1506-1512. 32228808 10.3201/eid2607.200574 PMC7323533

[bibr19-1087054720943271] Lu X. Zhang L. Du H. Zhang J. Li Y. Y. Qu J. Zhang W. Wang Y. Bao S. Li Y. Wu C. Liu H. Liu D. Shao J. Peng X. Yang Y. Liu Z. Xiang Y. Zhang F., . . . Wong G. W. K. (2020). SARS-CoV-2 infection in children. New England Journal of Medicine, 382(17), 1663–1665. 10.1056/NEJMc2005073 PMC7121177 32187458

[bibr20-1087054720943271] Pringle M. Ward P. Chilvers C. (1995). Assessment of the completeness and accuracy of computer medical records in four practices committed to recording data on computer. The British Journal of General Practice: The Journal of the Royal College of General Practitioners, 45(399), 537–541. 7492423 PMC1239405

[bibr21-1087054720943271] Shalev V. Chodick G. Goren I. Silber H. Kokia E. Heymann A. D. (2011). The use of an automated patient registry to manage and monitor cardiovascular conditions and related outcomes in a large health organization. International Journal of Cardiology, 152(3), 345–349. 20826019 10.1016/j.ijcard.2010.08.002

[bibr22-1087054720943271] Shim E. Tariq A. Choi W. Lee Y. Chowell G. (2020). Transmission potential and severity of COVID-19 in South Korea. International Journal of Infectious Diseases, 93, 339–344. 32198088 10.1016/j.ijid.2020.03.031 PMC7118661

[bibr23-1087054720943271] Sohrabi C. Alsafi Z. O’Neill N. Khan M. Kerwan A. Al-Jabir A. Losifidis C. Agha R. (2020). World Health Organization declares global emergency: A review of the 2019 novel coronavirus (COVID-19). International Journal of Surgery, 76, 71–76. 32112977 10.1016/j.ijsu.2020.02.034 PMC7105032

[bibr24-1087054720943271] Van Doremalen N. Bushmaker T. Morris D. H. Holbrook M. G. Gamble A. Williamson B. N. Williamson B. N. Tamin A. Harcourt J. L. Thornburg N. J. Gerber S. I. Lloyd-Smith J. O. de Wit E. Munster V. J. (2020). Aerosol and surface stability of SARS-CoV-2 as compared with SARS-CoV-1. New England Journal of Medicine, 382(16), 1564–1567. 10.1056/NEJMc2004973 PMC7121658 32182409

[bibr25-1087054720943271] World Health Organization. (2020a). Coronavirus disease (COVID-19): Situation report, 135. https://www.who.int/docs/default-source/coronaviruse/situation-reports/20200603-covid-19-sitrep-135.pdf?sfvrsn=39972feb_2 (accessed 4 June 2020).

[bibr26-1087054720943271] World Health Organization. (2020b). Coronavirus disease (COVID-19): Situation report, 12. https://www.who.int/docs/default-source/coronaviruse/situation-reports/20200201-sitrep-12-ncov.pdf?sfvrsn=273c5d35_2 (accessed 16 April 2020).

[bibr27-1087054720943271] World Health Organization. (2020c). Coronavirus disease (COVID-19) advice for the public. https://www.who.int/emergencies/diseases/novel-coronavirus-2019/advice-for-public (accessed 8 May 2020).

[bibr28-1087054720943271] World Health Organization (2020d). Global surveillance for human infection with coronavirus disease (COVID-2019). https://www.who.int/publicationsdetail/global-surveillance-for-human-infectionwith-novel-coronavirus-(2019-ncov) (accessed 4 June 2020).

[bibr29-1087054720943271] Wu F. Zhao S. Yu B. Chen Y.-M. Wang W. Song Z.-G. Hu Y. Tao Z.-W. Tian J.-H. Pei Y.-Y. Yuan M.-L. Zhang Y.-L. Dai F.-H. Liu Y. Wang Q.-M. Zheng J.-J. Xu L. Holmes E. C. Zhang Y.-Z. (2020). A new coronavirus associated with human respiratory disease in China. Nature, 579(7798), 265–269. https://doi:10.1038/s41586-020-2202-3 32015508 10.1038/s41586-020-2008-3 PMC7094943

